# Diagnostic Challenge of Mixed Lens-Induced Glaucoma After Chronic Traumatic Posterior Lens Dislocation: A Case Report

**DOI:** 10.3390/diagnostics16162545

**Published:** 2026-08-12

**Authors:** Tsu-Yuan Huang, Po-Chen Tseng, Chu-Yu Yen

**Affiliations:** 1 Department of Ophthalmology, Taipei City Hospital, Renai Branch, No. 10, Sec. 4, Ren’ai Rd., Da’an Dist., Taipei City 106243, Taiwan; 2 Department of Special Education, University of Taipei, No. 1, Aiguo W. Rd., Zhongzheng Dist., Taipei City 100234, Taiwan; 3 Department of Optometry, University of Kang-Ning, No. 137, Lane 75, Sec. 3, Kangning Rd., Neihu Dist., Taipei City 114311, Taiwan

**Keywords:** case report, lens dislocation, lens-induced glaucoma, phacolytic glaucoma, secondary glaucoma

## Abstract

**Background/Objectives:** Chronic traumatic posterior lens dislocation may cause ocular hypertension through overlapping lens-induced and traumatic mechanisms. We report a delayed presentation with a dislocated hypermature lens, a phacolytic phenotype, an incomplete capsule, and severe pressure elevation persisting after lens removal. **Case Presentation:** A 47-year-old man presented 5 years after blunt trauma with pain, redness, and blurred vision. Visual acuity was hand motion at 10 cm, and intraocular pressure (IOP) was 44.1 mmHg. Slit-lamp examination showed corneal edema, milky material in a deep anterior chamber, and the cataractous lens behind the iris. Ultrasonography localized the lens but could not assess capsular integrity or angle anatomy. Mixed lens-induced glaucoma with possible phacolytic, lens-particle, and traumatic components was diagnosed. Two days after 25-gauge pars plana vitrectomy and lensectomy, IOP remained 45.3 mmHg despite intensive therapy, prompting trabeculectomy with mitomycin C. At 6 months, IOP was 20.1 mmHg on three agents (qualified success), and vision remained hand motion at 30 cm with persistent corneal edema, aphakia, and suspected optic-nerve damage. **Conclusions:** Persistent IOP elevation after the removal of visible lens material suggested established trabecular or traumatic outflow dysfunction. Incomplete characterization of the capsule and angle precluded the assignment of a single mechanism.

## 1. Introduction

Lens-induced glaucoma encompasses several secondary glaucomas caused by the leakage of lens proteins, particulate obstruction, inflammation, or lens-related anatomic distortion. Phacolytic glaucoma typically presents with a mature or hypermature cataract, marked ocular hypertension, corneal edema, a deep anterior chamber, and a turbid or milky aqueous. Atypical presentations may require anterior-segment imaging or cytopathologic confirmation [1,2,3,4,5].

Prior trauma can obscure the underlying mechanism. A chronically subluxated or dislocated lens may become hypermature, release lens material, or develop an occult capsular defect that is not evident clinically. Phacolytic presentations have been reported with retained cortex behind the posterior capsule, dislocated hypermature lens nuclei, and lens dislocation accompanied by particles in the aqueous or vitreous [6,7,8]. Studies of traumatic lens dislocation support pars plana vitrectomy (PPV) with lensectomy when the lens has entered the posterior segment and describe associated retinal and pressure-related complications [9,10,11]. The literature on posteriorly displaced lens material also highlights the need to coordinate anterior- and posterior-segment management [12]. Separately, remote blunt trauma can cause angle recession and delayed glaucoma. When gonioscopy is limited, high-frequency ultrasound biomicroscopy (UBM) or anterior-segment optical coherence tomography (AS-OCT) may better define anterior-segment anatomy [13,14].

Here, we report a chronic traumatic posterior lens dislocation with a phacolytic phenotype and severe ocular hypertension that persisted after PPV and lensectomy. The clinically relevant feature was persistent pressure elevation after removal of the visible lens source, despite uncertainty about capsular integrity and angle anatomy. The delayed presentation, mixed clinical features, need for staged glaucoma surgery, and qualified rather than medication-free pressure control distinguish this course from uncomplicated lens-induced glaucoma that resolves after lens extraction.

## 2. Case Presentation

A 47-year-old man presented with pain, redness, and blurred vision in the right eye. Five years earlier, blunt trauma to that eye had resulted in traumatic lens dislocation. Vision had progressively declined over the preceding 6 months. He had no known drug allergies, asthma, or cardiac disease that would affect perioperative care or the use of glaucoma medications. The fellow eye had previously undergone phacoemulsification. Available records contained no history of trauma or glaucoma in the fellow eye and no documented bilateral angle abnormality.

**Figure 1:**
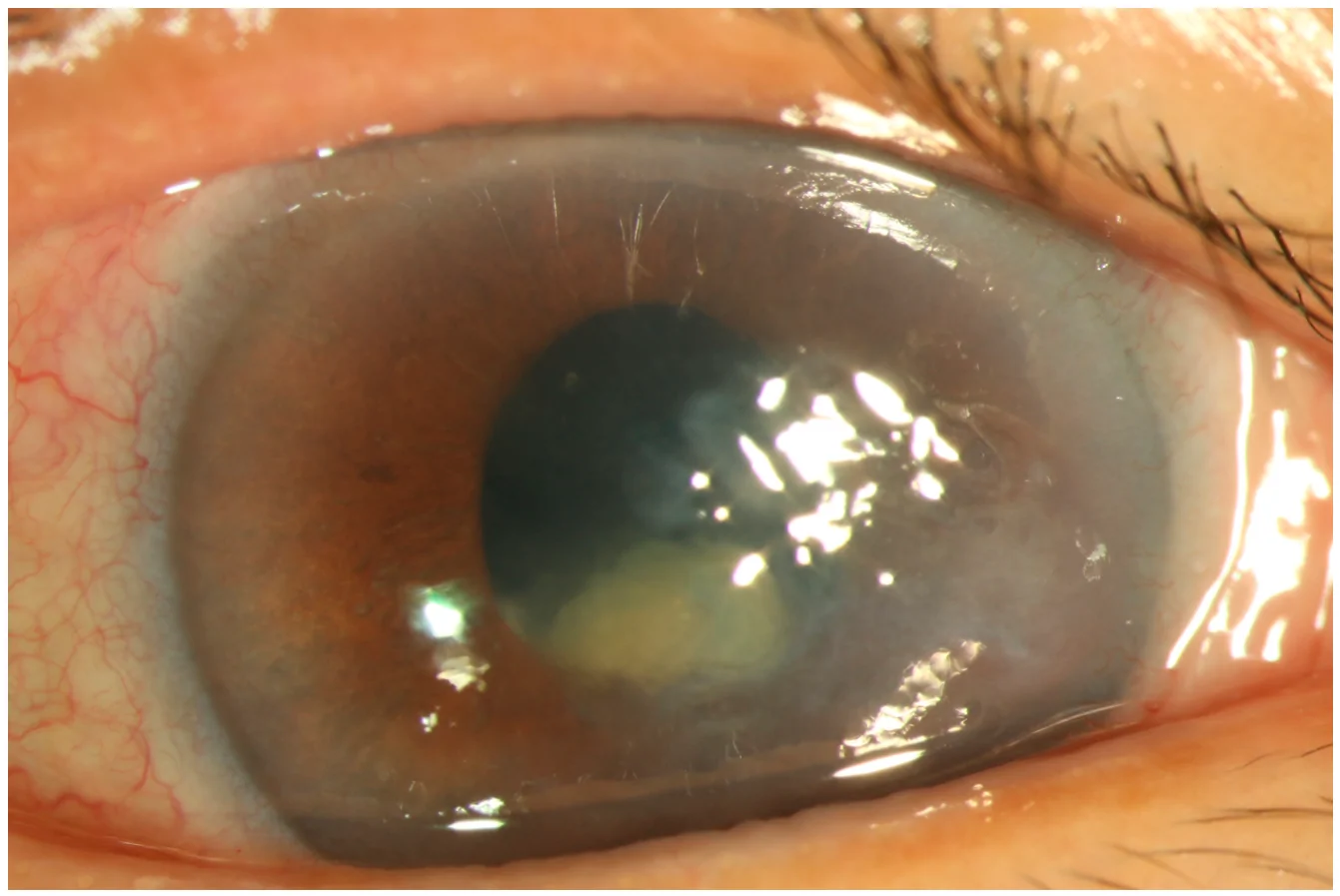
Slit-lamp appearance at presentation. The right eye showed corneal edema, turbid milky material in the anterior chamber, and a posteriorly dislocated hypermature cataractous lens.

**Figure 2:**
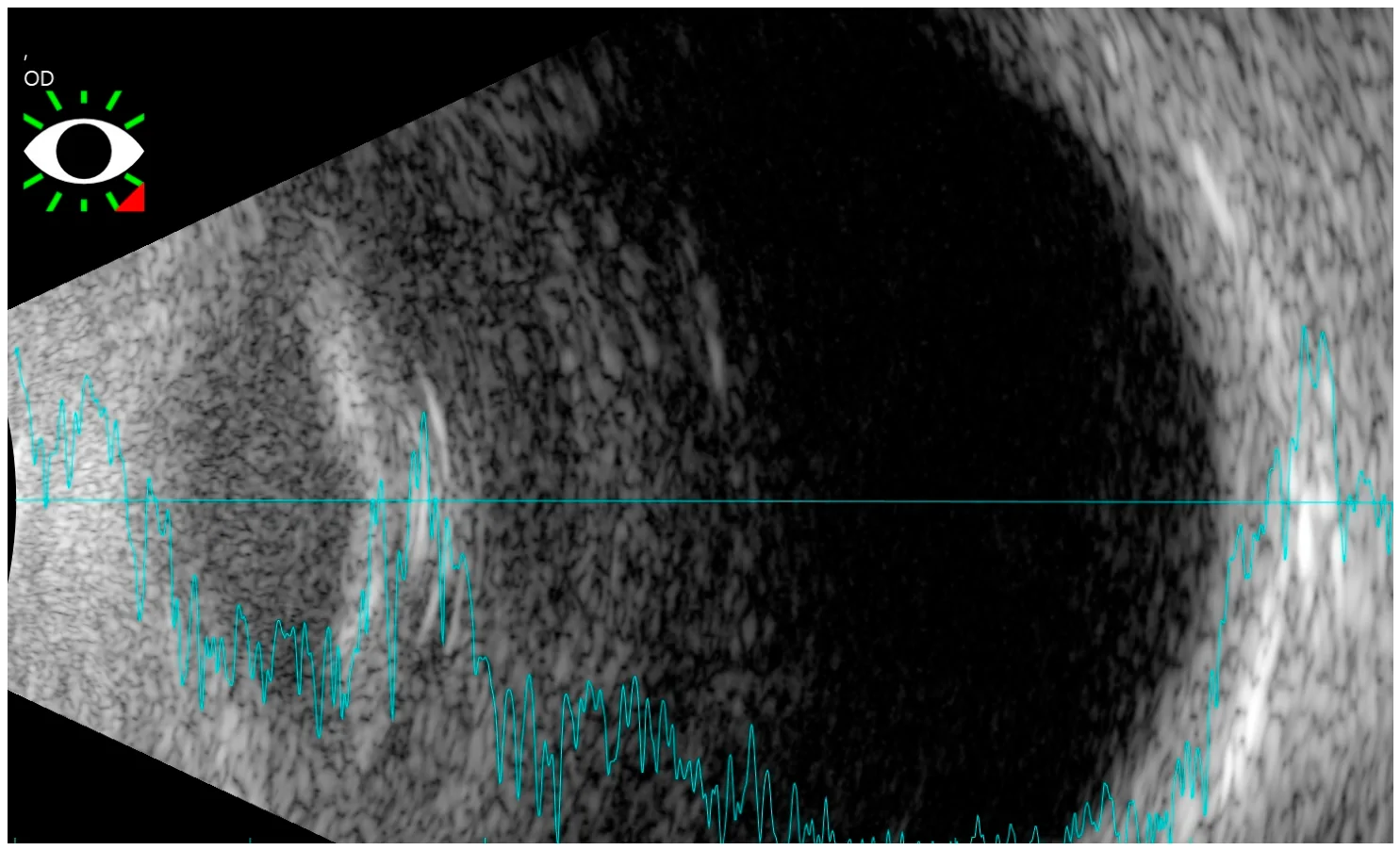
Conventional A- and B-scan ultrasonography of the right eye. The examination localized the posteriorly dislocated lens and showed no retinal detachment or intraocular mass. It was not used to assess capsular integrity or anterior-chamber angle anatomy.

At presentation, best-corrected visual acuity was hand motion (HM) at 10 cm in the right eye and 20/32 in the left eye. Noncontact air-puff tonometry did not produce a reliable reading in the right eye; all IOP values reported for that eye were obtained with an iCare IC100 rebound tonometer (Icare Finland Oy, Vantaa, Finland). IOP was 44.1 mmHg in the right eye and 13 mmHg in the left eye. Slit-lamp examination of the right eye showed conjunctival injection, localized corneal edema with Descemet membrane folds, and turbid, milky material with inflammatory cells in a deep anterior chamber. A cataractous lens was dislocated posteriorly behind the iris (Figure 1). There was no hypopyon, keratic precipitates, definite pigment dispersion, or polychromatic refractile crystals. Corneal edema limited gonioscopy; the visible angle segments appeared open, with no peripheral anterior synechiae. Repeat gonioscopy, UBM, and AS-OCT were not documented after stabilization, so the complete angle configuration could not be established. Media opacity also limited fundus examination. Conventional A- and B-scan ultrasonography localized the posteriorly dislocated lens and excluded retinal detachment and an intraocular mass (Figure 2).

Mixed lens-induced glaucoma with phacolytic features was the working diagnosis. The deep anterior chamber and milky inflammatory material suggested a predominantly open-angle lens-derived process, but incomplete gonioscopy and the absence of later high-resolution anterior-segment imaging precluded confirmation of a fully open angle. Classic phacolytic glaucoma involves leakage of soluble lens proteins without a clinically apparent major capsular rupture; in this traumatically dislocated lens, capsular integrity could not be confirmed preoperatively. Possible overlapping mechanisms, therefore, included a lens-particle component, angle recession, synechial change, and other traumatic damage to aqueous outflow. The differential diagnosis also included phacoantigenic inflammation, traumatic uveitis, infectious endophthalmitis, and synchysis scintillans. The absence of hypopyon and the lack of recent intraocular surgery, together with the chronic cataractous lens and milky rather than sparkling material, favored a lens-related inflammatory process.

Initial treatment included topical atropine 0.3% twice daily, brimonidine 0.15% three times daily, fixed-combination dorzolamide 2%/timolol 0.5% twice daily, and oral acetazolamide 250 mg four times daily. A prostaglandin analogue was avoided because of concerns that it might worsen ocular inflammation. Treatment was intended to lower IOP, control inflammation and ciliary spasm, and prepare the eye for removal of the posteriorly dislocated lens. Because the lens was in the posterior segment, the patient underwent 25-gauge PPV with fragmatome-assisted lensectomy rather than anterior-segment cataract extraction. The capsule was incomplete, although the location and extent of the defect and whether it predated surgery could not be determined. A small amount of free cortical material and an associated membranous reaction were present in the mid-vitreous. No obvious lens particles were seen in the anterior chamber, and no fragments adhered to the iris, ciliary body, or vitreous strands. All visible lens material was removed, with no residual cortex or capsular material seen at the end of surgery. Posterior-segment inspection showed an attached retina and a pale, cupped optic disc. These findings supported lens-particle and inflammatory components in addition to the phacolytic phenotype. Neither anterior-chamber fluid nor excised lens material was submitted for cytopathologic or histopathologic examination. Postoperative treatment comprised topical levofloxacin 0.5% and prednisolone acetate 1%, each four times daily; brimonidine, fixed-combination dorzolamide/timolol, and acetazolamide were continued at their preoperative doses.

Despite the removal of all visible lens material, IOP was 45.3 mmHg on post-lensectomy day 2 while the patient was receiving the full postoperative and pressure-lowering regimen described above. Because the severe pressure elevation persisted despite intensive medical therapy, fornix-based trabeculectomy with 0.04% mitomycin C (MMC; 0.4 mg/mL) was performed that day. A 3.5 × 3.5 mm square scleral flap was created. MMC was applied for 4 min and irrigated thoroughly, followed by punch sclerostomy and peripheral iridectomy.

**Figure 3:**
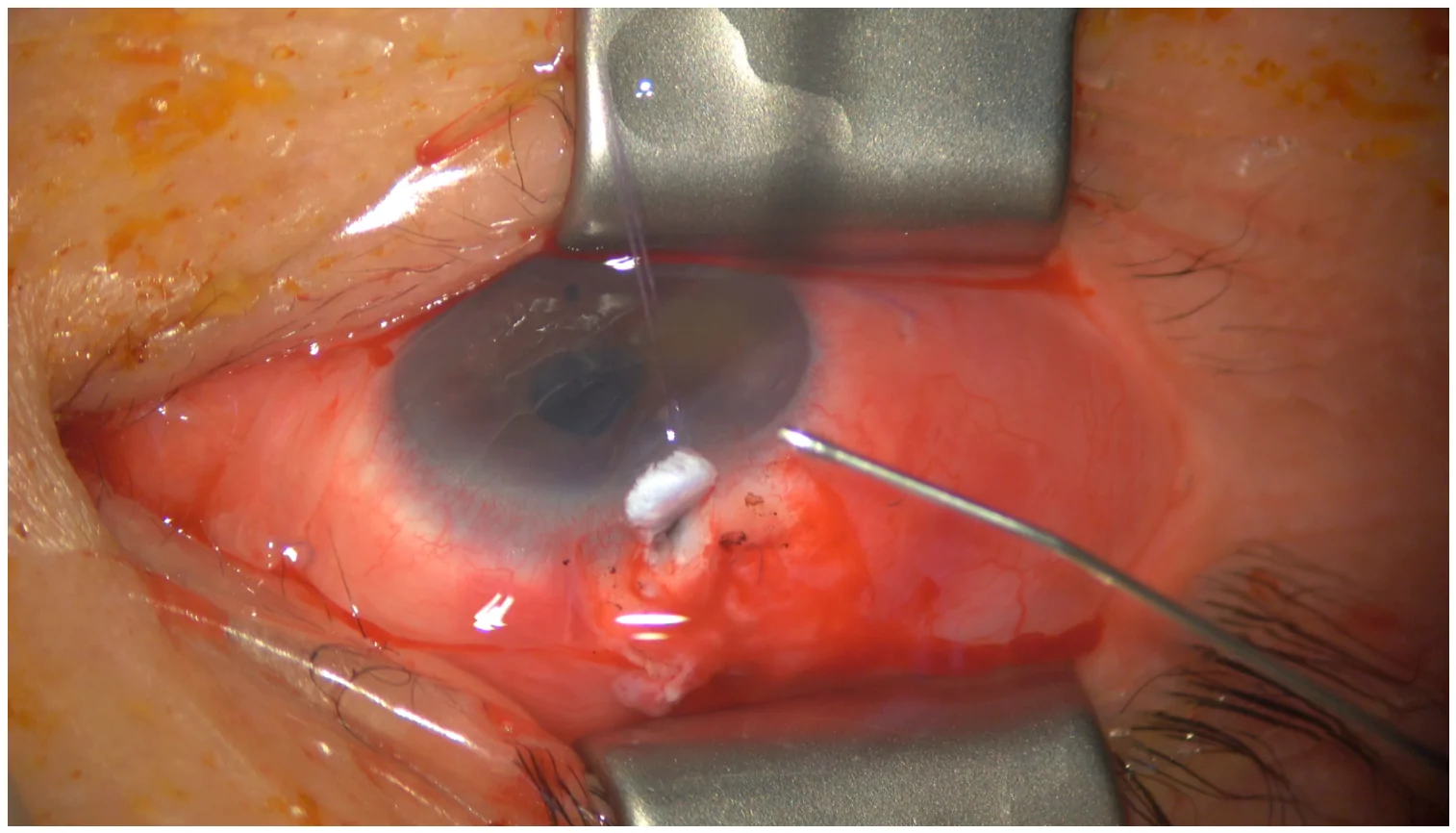
Intraoperative decompression during trabeculectomy. The photograph was obtained after punch sclerostomy and before scleral-flap suturing, when marked globe softening was noted clinically. The anterior chamber was subsequently reformed with approximately 0.1–0.2 mL of intracameral Provisc.

**Figure 4:**
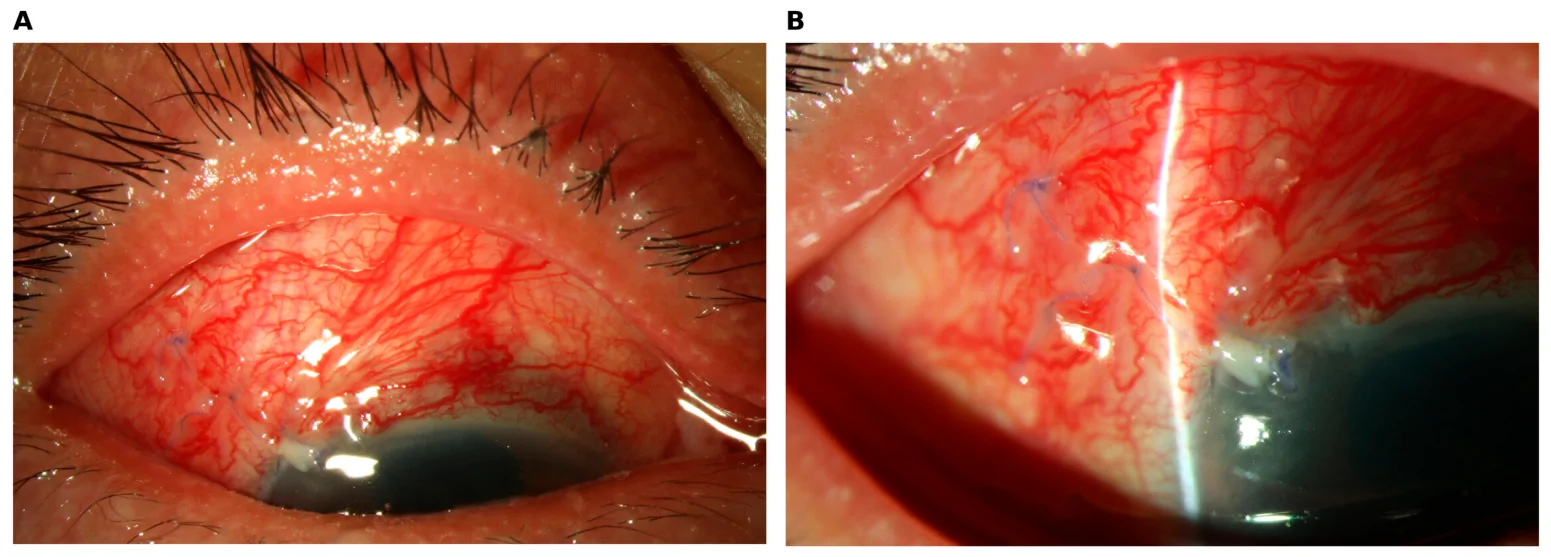
Early postoperative bleb appearance. Slit-lamp photographs obtained 6 days after trabeculectomy show diffuse conjunctival hyperemia (**A**) and an elevated superior filtering bleb (**B**).

**Figure 5:**
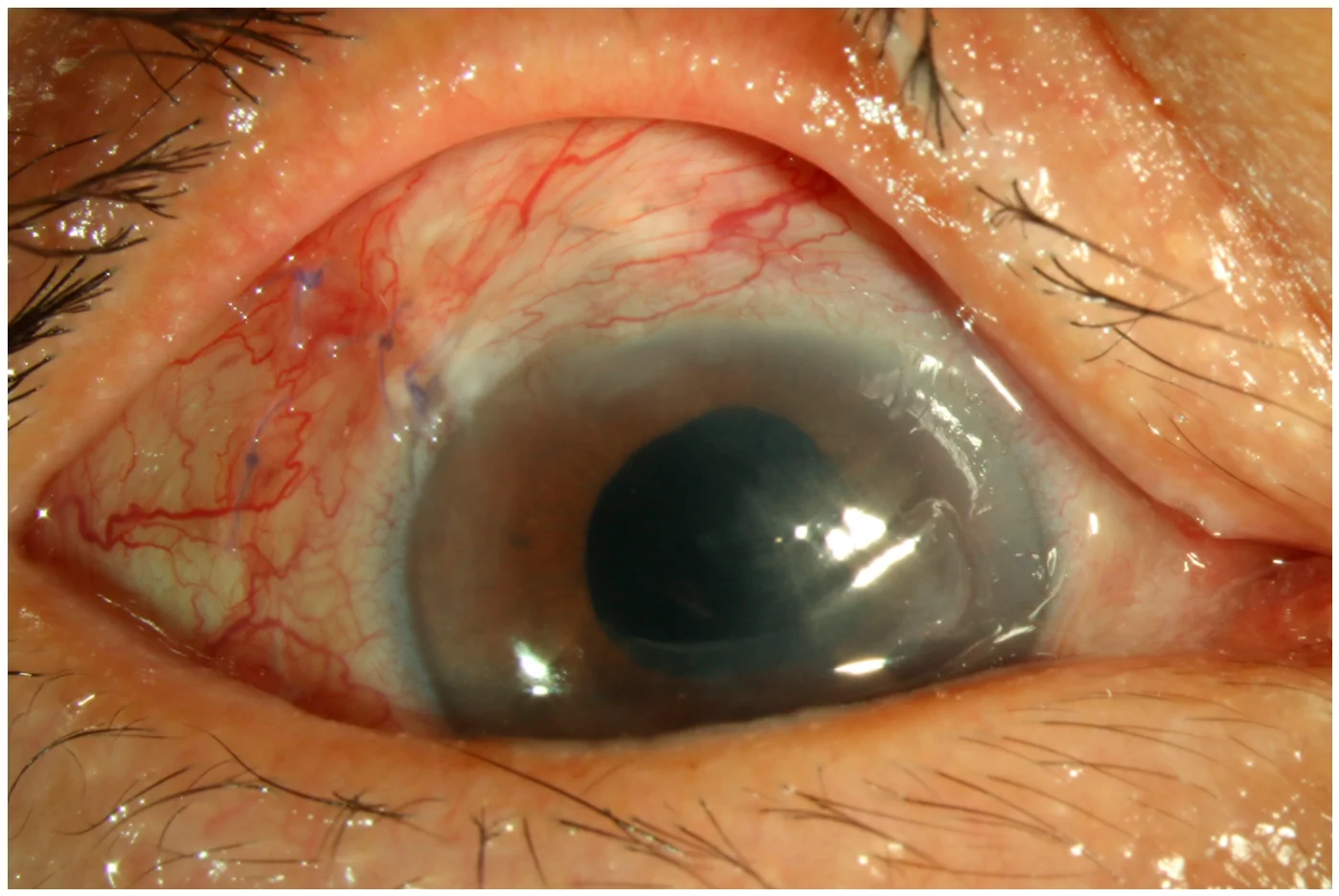
Slit-lamp appearance on postoperative day 20, showing persistent superior conjunctival injection after trabeculectomy.

After the sclerostomy and before scleral-flap suturing, the globe suddenly softened, consistent with the abrupt decompression of a previously vitrectomized eye (Figure 3). Approximately 0.1–0.2 mL of Provisc was injected into the anterior chamber to reform it; no anterior-chamber maintainer was used. Before closure, the viscoelastic was removed completely by irrigation with balanced salt solution. The anterior chamber was formed at the end of surgery. The scleral flap was secured with two fixed 10-0 nylon sutures, the conjunctiva was closed with 8-0 Vicryl, and the final Seidel test was negative. Brimonidine, fixed-combination dorzolamide/timolol, and acetazolamide were stopped on the day of the trabeculectomy. Levofloxacin 0.5% and prednisolone acetate 1%, each four times daily, were continued postoperatively. On day 6, IOP was 7 mmHg, and slit-lamp photographs showed diffuse conjunctival hyperemia and an elevated superior filtering bleb (Figure 4A,B). At 2 weeks, IOP had risen to 35 mmHg. Approximately 5 s of physician-performed digital massage reduced it to 24 mmHg, and the patient was instructed to continue digital massage at home. Brimonidine 0.15% three times daily and fixed-combination dorzolamide 2%/timolol 0.5% twice daily were restarted; acetazolamide was not used thereafter. Levofloxacin was continued for 1 month after trabeculectomy and then stopped. Prednisolone acetate was reduced from four to two times daily at 2 weeks because of concerns about a possible steroid response, while early bleb scarring remained a competing risk; it was stopped at 1 month. On postoperative day 20, IOP was 20.3 mmHg before massage, and superior conjunctival injection persisted (Figure 5). Because IOP responded to massage and improved after topical therapy was restarted, no laser suture lysis, bleb needling, subconjunctival 5-fluorouracil injection, or other bleb intervention was performed during follow-up.

Six months after trabeculectomy, persistent corneal edema precluded formal refraction. Visual acuity remained HM at 30 cm and did not improve with pinhole or a high-plus aphakic trial lens. The prognosis was guarded because of the persistent edema and aphakia; the pale, cupped optic disc observed intraoperatively also raised concerns about irreversible glaucomatous or traumatic optic-nerve damage, although quantitative structural and functional testing was unavailable. IOP was 20.1 mmHg on brimonidine 0.15% three times daily and fixed-combination dorzolamide 2%/timolol 0.5% twice daily, which represented three active agents in two bottles. By the criteria defined in the Discussion, this was a qualified rather than complete success. The anterior chamber and vitreous were quiet, but the corneal edema was unchanged. No clinical evidence of retinal detachment, choroidal detachment, persistent hypotony, blebitis, or endophthalmitis was documented after surgery. Posterior-segment follow-up remained limited by media opacity; the retina had been attached at intraoperative inspection. Secondary intraocular lens implantation was deferred because of corneal edema, unstable glaucoma status, and limited visual potential. Direct fundus examination, optic-nerve imaging, and visual-field testing would be reconsidered if media clarity and visual function improved. Table 1 summarizes the clinical course.

During follow-up, the patient acknowledged that the delayed presentation after the traumatic lens dislocation had contributed to the severe ocular hypertension and limited visual recovery. He accepted the need for staged surgery, continued topical glaucoma therapy, and long-term follow-up and reported no major difficulty attending visits or using the prescribed medications. The CARE checklist is provided as Supplementary File S1.

## 3. Discussion

Secondary glaucoma after lens dislocation is well recognized, but this patient had an unusual sequence: a chronically posteriorly dislocated hypermature lens with a phacolytic phenotype; incomplete characterization of the capsule and angle; IOP of 45.3 mmHg 2 days after removal of all visible lens material; and only qualified pressure control after trabeculectomy. Lens removal was necessary, but the failure of IOP to normalize was consistent with established trabecular or traumatic outflow damage.

Mechanistic classification remains uncertain. Lens particles, soluble high-molecular-weight lens proteins, and inflammatory cells can obstruct aqueous outflow [15]. Cytopathologic and imaging studies indicate that clinical appearance alone may not distinguish phacolytic glaucoma from lens-particle and other lens-induced mechanisms [2,5,6,7,8]. In this patient, the capsule was incomplete, but the site, extent, and timing of the defect could not be established. Free cortical material and a membranous reaction in the mid-vitreous supported lens-particle and inflammatory components. No lens particles were visible in the anterior chamber; no fragments adhered to the iris, ciliary body, or vitreous strands; and no residual cortex or capsular material was seen after lensectomy. Cytology and histopathology were not obtained, and angle anatomy remained incompletely characterized. Accordingly, we classified the condition as mixed lens-induced glaucoma with phacolytic features and possible lens-particle and traumatic overlap, rather than isolated phacolytic glaucoma.

Infectious endophthalmitis was considered but was less likely because there had been no recent intraocular procedure in the affected eye and no hypopyon; the chronic cataractous lens and milky anterior chamber better supported a lens-related process. Phacoantigenic inflammation and traumatic uveitis may have contributed but did not fully account for the marked pressure elevation. Synchysis scintillans produces highly refractile cholesterol crystals in chronically damaged eyes [16], whereas no polychromatic sparkling crystals were documented. Traumatic glaucoma remained plausible because remote blunt injury can cause angle recession and delayed outflow failure [13]. Because the complete angle was not documented after initial stabilization, angle recession, peripheral anterior synechiae, and partial synechial closure could not be excluded.

Conventional ophthalmic ultrasonography was useful for locating the posteriorly displaced lens and screening for retinal detachment, vitreous abnormalities, and an intraocular mass. It does not provide the microscopic anterior-segment resolution required to identify a capsular defect, determine the relationship between lens material and the trabecular meshwork, or define the angle. High-frequency UBM, commonly performed at approximately 50–100 MHz, can image the iris, ciliary body, lens, and angle through opaque media; AS-OCT provides complementary noncontact assessment when corneal clarity permits [5,14]. The absence of repeat gonioscopy, UBM, and AS-OCT prevented more precise mechanistic classification.

Lens location dictated the surgical approach. For a cataractous crystalline lens in the posterior segment, PPV permits controlled lensectomy, management of vitreoretinal traction, and inspection or treatment of posterior-segment complications. Studies of traumatic lens dislocation support PPV with lensectomy and show that associated trauma, retinal breaks, and postoperative pressure abnormalities influence visual outcomes [9,11]. The literature on posteriorly displaced lens material also supports coordinated anterior- and posterior-segment planning [12]. Intraoperative inspection in this case showed an attached retina and a pale, cupped optic disc. The disc appearance was consistent with limited visual potential, although OCT and visual-field testing were unavailable. Studies of retained postoperative lens fragments are not directly comparable with traumatic whole-lens dislocation, but they document the inflammatory and pressure-related effects of posterior lens material and the role of pars plana removal [17,18].

The persistence of severe ocular hypertension after lensectomy was clinically important. Lens removal stops further release of proteins or particles but cannot immediately reverse macrophage-mediated obstruction, inflammatory trabecular injury, angle recession, or synechial change. In recent cohorts, most eyes with lens-induced glaucoma had substantial IOP reduction after timely lens surgery; delayed presentation was associated with poorer visual outcomes, and some eyes required continued glaucoma therapy [19,20,21]. An IOP of 45.3 mmHg 2 days after the complete removal of the visible lens material was, therefore, unlikely to reflect a transient lens-source effect alone. A prostaglandin analogue had been withheld because of concerns about inflammation, but IOP remained extreme despite brimonidine, fixed-combination dorzolamide/timolol, and systemic acetazolamide.

Trabeculectomy achieved a qualified, not complete, success. A recent series of traumatic lens dislocations with secondary glaucoma reported pressure control after combined lentectomy, vitrectomy, and trabeculectomy but also described hypotony, choroidal detachment, hemorrhage, and hyperfiltration [10]. In a large trabeculectomy cohort, complete success was defined as IOP 4–21 mmHg without medication and qualified success as the same IOP range with medication [22]. At 6 months, the present eye had an IOP of 20.1 mmHg on three agents and, therefore, met the criterion for qualified but not complete success. The early rebound to 35 mmHg and continued medication requirement indicated limited bleb function or residual distal resistance and warranted ongoing surveillance.

IOP fell to 7 mmHg by day 6, indicating early filtration, but rose to 35 mmHg at 2 weeks. Possible contributors included early subconjunctival fibrosis, limited flap flow, a steroid-related pressure response, and distal outflow resistance. Five seconds of physician-performed digital massage lowered IOP from 35 to 24 mmHg, which suggests that resistance at the bleb or flap remained partly modifiable. Brimonidine and fixed-combination dorzolamide/timolol were restarted, and the patient was instructed to perform massage at home. Prednisolone acetate was reduced from four to two times daily at 2 weeks to balance concerns about a steroid response against the need to suppress early inflammation and scarring; it was discontinued at 1 month. Levofloxacin was also discontinued at 1 month. By postoperative day 20, IOP was 20.3 mmHg before massage. The immediate response to massage and subsequent improvement after the resumption of topical therapy supported observation without laser suture lysis, bleb needling, subconjunctival 5-fluorouracil injection, or another bleb intervention. During trabeculectomy, abrupt decompression after sclerostomy was managed by reforming the anterior chamber with approximately 0.1–0.2 mL of intracameral Provisc. The viscoelastic was removed before closure, the flap was secured with two fixed 10-0 nylon sutures, the conjunctiva was closed with 8-0 Vicryl, and the final Seidel test was negative.

Limitations include the single-patient design and incomplete anatomic, pathologic, and functional assessment. Repeat gonioscopy, UBM, and AS-OCT were not documented, so the angle could not be fully characterized. Although the capsule was incomplete and free cortical material with a membranous reaction was found in the mid-vitreous, the site, extent, and timing of the capsular defect remained unknown, and no specimen was submitted for cytology or histopathology. Persistent corneal edema prevented meaningful fundus examination, OCT, perimetry, and formal refraction at 6 months; only qualitative intraoperative findings of an attached retina and a pale, cupped optic disc were available. Longer-term pressure, bleb survival, and visual data were also unavailable. These limitations prevent precise separation of phacolytic, lens-particle, inflammatory, and traumatic mechanisms. Nevertheless, the persistence of extreme IOP after the removal of all visible lens material, followed by qualified control after trabeculectomy, supports established outflow dysfunction.

## 4. Conclusions

Chronic traumatic posterior lens dislocation can produce mixed lens-induced glaucoma with phacolytic, lens-particle, and traumatic components. When gonioscopy, high-resolution anterior-segment imaging, and pathology are incomplete, a single mechanism should not be assigned. In this patient, severe ocular hypertension persisted after lensectomy, which suggested established outflow dysfunction and required trabeculectomy. At 6 months, pressure control was qualified rather than medication-free, and the visual prognosis remained guarded because of persistent corneal edema, aphakia, and suspected irreversible optic-nerve damage.

## Figures and Tables

**Table 1:** Clinical course, treatment, and intraocular pressure.

Clinical Timeline	Key Findings	Management and Interpretation	Medication Status
Presentation	Right-eye pain, redness, blurred vision; HM at 10 cm; IOP 44.1 mmHg by iCare IC100 rebound tonometry after noncontact air-puff tonometry did not yield a reliable reading; corneal edema; deep turbid anterior chamber; posteriorly dislocated cataractous lens; angle assessment incomplete.	Mixed lens-induced glaucoma with phacolytic features was suspected; the complete angle configuration could not be confirmed.	Atropine 0.3% twice daily; brimonidine 0.15% three times daily; dorzolamide 2%/timolol 0.5% twice daily; acetazolamide 250 mg four times daily. A prostaglandin analogue was avoided because of concerns about exacerbating ocular inflammation.
Definitive lens surgery	Posteriorly dislocated hypermature cataractous lens; incomplete capsule of indeterminate location, extent, and timing; small amount of free cortical material in the mid-vitreous; membranous reaction; no obvious anterior-chamber lens particles or fragment adherence; retina attached and optic disc pale/cupped on intraoperative inspection.	25-gauge PPV with fragmatome-assisted lensectomy; all visible lens material was removed, with no visible residual cortex or capsular material; no cytology or histopathology was obtained.	Levofloxacin 0.5% four times daily and prednisolone acetate 1% four times daily postoperatively; brimonidine 0.15% three times daily, dorzolamide 2%/timolol 0.5% twice daily, and acetazolamide 250 mg four times daily continued.
Post-lensectomy day 2	IOP 45.3 mmHg despite an intensive multidrug pressure-lowering regimen; prostaglandin analogue avoided because of concern for inflammation.	Persistent severe ocular hypertension suggested residual outflow dysfunction; trabeculectomy was performed on post-lensectomy day 2.	Levofloxacin 0.5% four times daily and prednisolone acetate 1% four times daily; brimonidine 0.15% three times daily; dorzolamide 2%/timolol 0.5% twice daily; acetazolamide 250 mg four times daily.
Trabeculectomy	Fornix-based trabeculectomy on post-lensectomy day 2 with 0.04% MMC; 3.5 × 3.5 mm square scleral flap; transient intraoperative globe softening after sclerostomy.	Approximately 0.1–0.2 mL intracameral Provisc was used to reform the anterior chamber; no anterior-chamber maintainer was used. Provisc was completely removed with balanced salt solution. The flap was secured with two fixed 10-0 nylon sutures, the conjunctiva was closed with 8-0 Vicryl, the chamber was formed, and the Seidel test was negative.	Brimonidine, dorzolamide 2%/timolol 0.5%, and acetazolamide were discontinued on the day of trabeculectomy. Levofloxacin 0.5% four times daily and prednisolone acetate 1% four times daily were prescribed postoperatively.
Trabeculectomy day 6	IOP 7 mmHg; conjunctival hyperemia and elevated superior filtering bleb.	Early filtration effect was observed; subsequent treatment was adjusted according to IOP response.	Levofloxacin 0.5% four times daily and prednisolone acetate 1% four times daily; pressure-lowering agents remained discontinued.
2 weeks after trabeculectomy	IOP 35 mmHg, reduced to 24 mmHg after approximately 5 s of physician-performed digital massage.	Digital massage produced an immediate pressure response; the patient was instructed to perform massage at home. Brimonidine and fixed-combination dorzolamide/timolol were restarted. Prednisolone was reduced to twice daily to balance concern about a possible steroid response against the risk of early bleb scarring.	Levofloxacin 0.5% four times daily; prednisolone acetate 1% reduced from four times daily to twice daily; brimonidine 0.15% three times daily and dorzolamide 2%/timolol 0.5% twice daily restarted. Acetazolamide was not included thereafter.
Trabeculectomy day 20	IOP 20.3 mmHg before massage; persistent superior conjunctival injection on slit-lamp photography.	Because IOP responded to digital massage and improved to 20.3 mmHg after topical therapy was restarted, no laser suture lysis, bleb needling, subconjunctival 5-fluorouracil injection, or other postoperative bleb intervention was performed.	Levofloxacin 0.5% four times daily; prednisolone acetate 1% twice daily; brimonidine 0.15% three times daily; dorzolamide 2%/timolol 0.5% twice daily.
Trabeculectomy month 6	IOP 20.1 mmHg; HM at 30 cm without improvement through pinhole or high-plus aphakic trial-lens testing; persistent corneal edema unchanged; aphakia; anterior chamber and vitreous quiet; no postoperative clinical evidence of retinal detachment, choroidal detachment, persistent hypotony, blebitis, or endophthalmitis.	Qualified rather than complete success based on published criteria; levofloxacin and prednisolone were stopped at month 1; secondary intraocular lens implantation was deferred; visual prognosis remained guarded because of persistent corneal edema, aphakia, and the pale, cupped optic disc seen intraoperatively.	Brimonidine 0.15% three times daily; dorzolamide 2%/timolol 0.5% twice daily (three active agents in two bottles).

Abbreviations: HM, hand motion; IOP, intraocular pressure; MMC, mitomycin C; PPV, pars plana vitrectomy. All reported IOP measurements were obtained with an iCare IC100 rebound tonometer (Icare Finland Oy, Vantaa, Finland) because noncontact air-puff tonometry did not produce a reliable reading in the affected eye.

## Data Availability

The original contributions presented in this study are included in the article and Supplementary Material. Additional patient-level data are not publicly available because of privacy and consent restrictions. Further inquiries can be directed to the corresponding author, subject to institutional approval.

## Supplementary Materials

The following supporting information can be downloaded at: https://www.mdpi.com/article/10.3390/diagnostics16162545/s1, Supplementary File S1: CARE case report checklist with manuscript locations.

## Abbreviations

HM, hand motion; IOP, intraocular pressure; MMC, mitomycin C; PPV, pars plana vitrectomy.
