# Supplementary material for: Diagnostic Challenge of Mixed Lens-Induced Glaucoma After Chronic Traumatic Posterior Lens Dislocation: A Case Report
Source: Diagnostics (Basel). 2026 Aug 12;16(16):2545. doi: 10.3390/diagnostics16162545 (PMC13512798; doi:10.3390/diagnostics16162545)
Supplement: Supplementary file 1 [file diagnostics-16-02545-s001.zip › diagnostics-4454239-supplementary.pdf]

CARE Checklist

Diagnostic Challenge of Mixed Lens-Induced Glaucoma after Chronic Traumatic Posterior Lens Dislocation: A Case Report

Supplementary File S1. CARE case report checklist with manuscript locations.

CARE item	Topic	Checklist requirement	Location in manuscript
1	Title	The diagnosis or focus is followed by the words "case report".	Title page
2	Keywords	Keywords identify the main diagnoses and include "case report".	Keywords section
3	Abstract	Abstract includes novelty, patient presentation, diagnosis, interventions, outcomes, and take-away conclusion.	Abstract: Background and Clinical Significance; Case Presentation; Conclusions
4	Introduction	Explains why chronic traumatic posterior lens dislocation with a phacolytic phenotype and persistent ocular hypertension is unusual.	1. Introduction
5	Patient information	De-identified demographic information, presenting symptoms, ocular trauma history, relevant medical history, family/psychosocial history, and fellow-eye history are reported.	2. Case Presentation, opening paragraphs
6	Clinical findings	Visual acuity, intraocular pressure, slit-lamp findings, gonioscopy limitation, and ultrasonographic findings are described.	2. Case Presentation; Figures 1 and 2
7	Timeline	Clinical course and intraocular pressure changes are organized in a timeline table.	Table 1
8	Diagnostic assessment	Diagnostic reasoning, ultrasonography, differential diagnoses, diagnostic uncertainty, and lack of cytopathology/histopathology confirmation are described.	2. Case Presentation; 3. Discussion
9	Therapeutic intervention	Medical therapy, pars plana vitrectomy with fragmatome-assisted lensectomy, and trabeculectomy with mitomycin C are reported with key doses and procedural details.	2. Case Presentation; Table 1
10	Follow-up and outcomes	Postoperative pressure course, 6-month findings, complications, medication status, visual outcome, and adherence are reported.	2. Case Presentation; Table 1
11	Discussion	Diagnostic overlap, literature context, rationale for conclusions, limitations, and primary clinical lesson are discussed.	3. Discussion; 4. Conclusions
12	Patient perspective	Patient understanding of delayed presentation, limited visual recovery, staged treatment, medication use, and follow-up is summarized.	2. Case Presentation, patient-perspective paragraph
13	Informed consent	Written informed consent for publication of clinical details and accompanying images is stated.	Informed Consent Statement
